# Laparoscopic Sigmoidectomy in a Male Colon Cancer Patient With Pelvic Arteriovenous Malformation Using Preoperative Interventional Radiology: A Case Report

**DOI:** 10.1111/ases.70037

**Published:** 2025-02-20

**Authors:** Gaku Inaguma, Koki Otsuka, Koji Masumori, Junichiro Hiro, Tsutomu Kumamoto, Megumu Kamishima, Yosuke Kobayashi, Yongchol Chong, Yusuke Omura, Hiroko Taniguchi, Kazuki Tsujimura, Yuko Chikaishi, Ayako Tsurumachi, Hokuto Akamatsu, Ichiro Uyama, Koichi Suda

**Affiliations:** ^1^ Department of Surgery Fujita Health University Toyoake Japan; ^2^ Department of Advanced Robotic and Endoscopic Surgery, School of Medicine Fujita Health University Toyoake Japan; ^3^ Department of Radiology Fujita Health University Toyoake Japan; ^4^ Collaborative Laboratory for Research and Development in Advanced Surgical Technology Fujita Health University Toyoake Japan; ^5^ Collaborative Laboratory for Research and Development in Advanced Surgical Intelligence Fujita Health University Toyoake Japan

**Keywords:** arteriovenous malformations, interventional, laparoscopyradiologysigmoid neoplasms

## Abstract

Pelvic arteriovenous malformation (AVM) is a rare vascular condition with diverse clinical manifestations. Treatment‐related decision‐making is difficult for concurrent AVMs and colon cancer. Interventional radiology is effective for colon cancer patients with pelvic AVM. Herein, a 77‐year‐old man presented with fatigue. Computed tomography revealed thickening of the sigmoid colon wall without lymph node swelling or distant metastasis, confirming irregularly dilated pelvic blood vessels. Preoperative transcatheter embolization of the AVM was initially performed. Then, laparoscopic sigmoidectomy was performed without complications following confirmation of AVM shrinkage via computed tomography. The patient was discharged without complications. Thus, preoperative pelvic AVM embolization in patients with sigmoid colon cancer may facilitate safe minimally invasive surgery.

## 1 Introduction

Arteriovenous malformations (AVMs) are rare vascular conditions in which the arterial and venous systems are abnormally connected without a normal intervening capillary network [1]. While most AVMs are congenital, they can also arise after surgery or trauma [1]. Clinically, the manifestations of this condition range from large asymptomatic vascular lesions observed on pelvic imaging to life‐threatening bleeding and congestive heart failure [2]. Pelvic AVMs occur in < 1% of the general population and are, particularly, rare in male patients [3]. In recent years, interventional radiology (IVR), an alternative to resection, has shown favorable outcomes [4].

Colorectal cancer, the third most common cancer worldwide, is one of the leading causes of mortality [5]. In Western countries, laparoscopic surgery for colorectal cancer is accepted as the gold standard and is associated with better postoperative outcomes than open surgery [6]. In Japan, the short‐term outcomes of minimally invasive surgery for colon cancer are more acceptable than those of open surgery [7].

There are no reports of laparoscopic colectomy performed in male colon cancer patients with pelvic AVM. Herein, we report our experience in performing a safe laparoscopic colectomy for one such patient using preoperative IVR to reduce the risk of bleeding due to AVM injury.

## 2 Case Presentation

**FIGURE 1:**
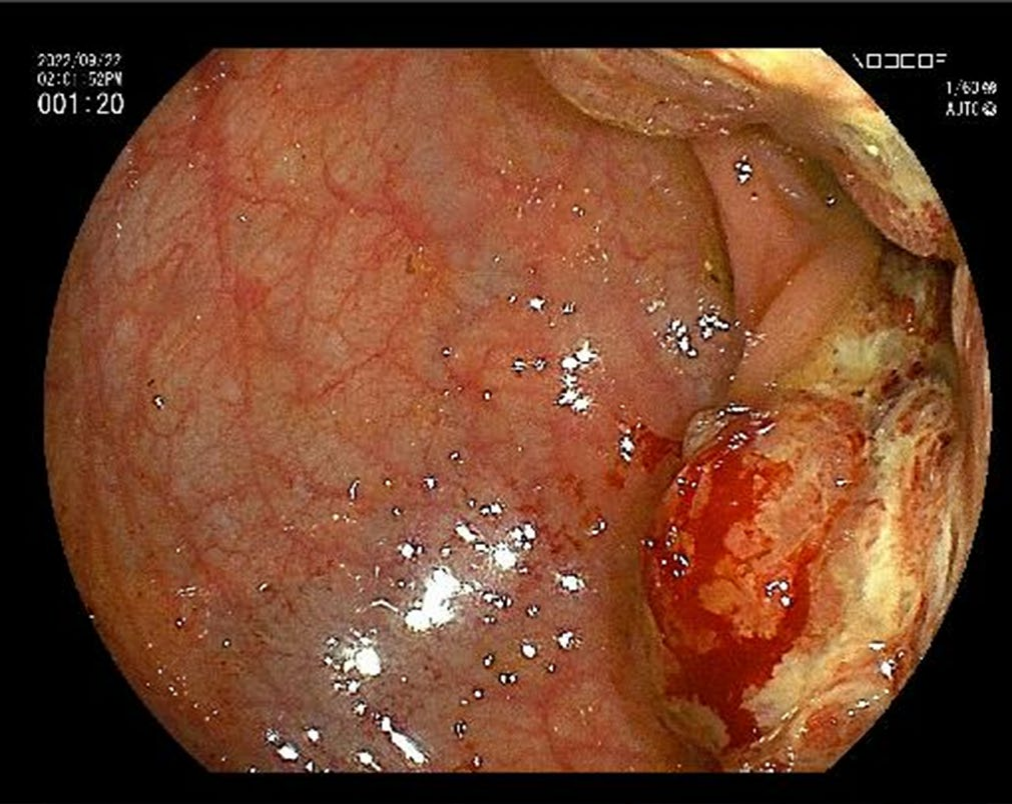
Colonoscopy image. Colonoscopy revealed an ulcerative lesion with clear margins in the sigmoid colon.

**FIGURE 2:**
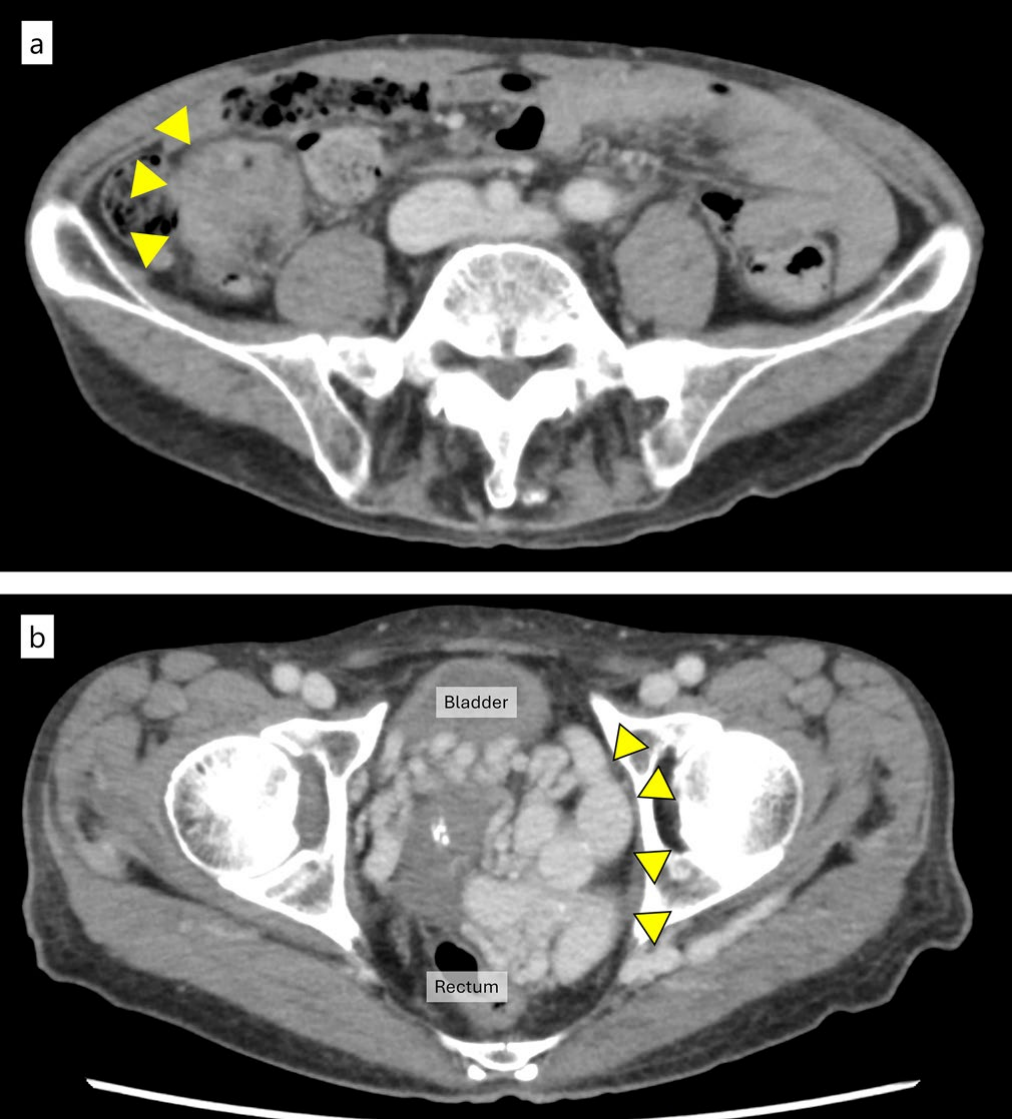
Computed tomography images before surgery. (a) The sigmoid colon was long, and the tumor was located on the right side of its body (yellow arrowheads). There was no obvious lymph node swelling or distant metastasis. (b) Dilated blood vessels are seen on the left side of the pelvis (yellow arrowheads).

A 77‐year‐old man presented with fatigue lasting 2 months prior to consultation. The patient had no medical or family history of vascular malformations. Laboratory tests revealed unremarkable findings, except for anemia (hemoglobin concentration, 11.1 g/dL) and elevated carcinoembryonic antigen levels (6.1 ng/mL). Colonoscopy revealed a Type 2 mass in the sigmoid colon (Figure 1). Computed tomography (CT) revealed thickening of the sigmoid colon wall without lymph node swelling or distant metastasis (Figure 2a), confirming irregularly dilated pelvic blood vessels (Figure 2b). Biopsy with histopathology revealed adenocarcinoma. Therefore, the clinical diagnosis based on the tumor's TNM classification was confirmed as cT3, cN0, cM0, cStage IIA cancer.

**FIGURE 3:**
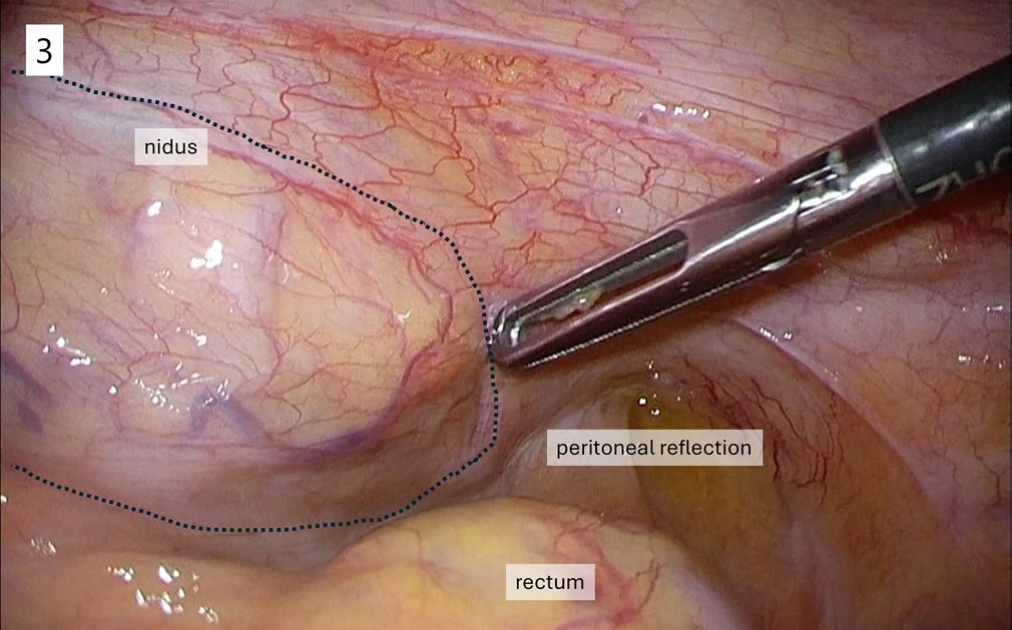
Intraoperative image of the pelvis. The nidus (within the dotted line) protruded into the abdominal cavity from the left retroperitoneum of the pelvis.

Pelvic three‐dimensional CT angiography revealed an AVM with the left internal iliac artery as the inflow artery, a nidus (dilated abnormal vessel), and the right great saphenous vein as the outflow vein (Figures S1 and S2). Pelvic angiography revealed that the inflow artery was the left obturator artery, a branch of the left internal iliac artery (Video S3). Preoperative transcatheter embolization of the inflow artery and outflow vein was performed to prevent intraoperative bleeding due to AVM injury. The inflow artery was embolized transarterially with *n*‐butyl‐2‐cyanoacrylate (NBCA). Despite this, microinflow arteries and veins from the nidus were still visible (Video S4), necessitating embolization of the outflow veins. The outflow vein was embolized percutaneously with a penumbra coil (PC 400; Penumbra Inc., Alameda, CA, USA) and a hydrogel coil (Azur; Terumo Corp., Tokyo, Japan) (Videos S5 and S6). However, the embolization was insufficient, and NBCA was added to ensure complete embolization. CT performed 3 weeks after embolization revealed shrinkage and thrombosis of the nidus (Figure S7); thus, laparoscopic sigmoidectomy was performed (Video S8). During rectal examination prior to surgery, the thrombus‐forming nidus was palpated as a solid mass on the left side of the rectum. Therefore, D3 lymph node dissection was performed, and the inferior mesenteric artery was ligated at its root. The sigmoid colon mesentery was mobilized from the retroperitoneum, and the tumor was extracted via the umbilical incision. After the anvil head was attached to the descending colon, the colon was returned to the abdominal cavity. A sizer was used to simulate device insertion before placing the anastomotic device. Finally, the anastomotic device was carefully inserted transanally, and end‐to‐end anastomosis was performed using the double‐stapling technique with a 25‐mm circular stapler (Figure 3). The surgery duration was 128 min, and the estimated blood loss was 11 mL. The patient had no postoperative complications and was discharged 6 days after surgery.

Histological analyses revealed mucinous and moderately differentiated adenocarcinomas. The tumor was exposed to the serosa. Lymph node metastasis was observed in one lymph node. The distal and proximal surgical margins were 100 and 90 mm, respectively (Figure S9). The pathological stage of the tumor was pT4a, pN1a, cM0, and pStage IIIb. Postoperative adjuvant chemotherapy was not performed. Pulmonary recurrence was observed 6 months after surgery. Thoracoscopic partial pneumonectomy was performed 8 months after surgery. No recurrence was observed at 12 months after pulmonary surgery.

## 3 Discussion

AVMs are rare congenital vascular diseases in which the arterial and venous systems connect in the absence of a normal capillary network [1]. The prevalence of pelvic AVM is reported to be < 1% in the general population, and it is particularly rare in male patients [3]. In cases where colorectal cancer is not present, asymptomatic pelvic AVMs are typically managed with observation, as supported by several follow‐up studies [4, 8]. This approach minimizes the risks associated with unnecessary procedures. However, in this case, the presence of sigmoid colon cancer necessitated preoperative embolization to reduce the risk of intraoperative bleeding and ensure the safety of the surgical procedure. The decision to treat pelvic AVMs should be individualized, considering the patient's overall clinical background and the risks associated with the condition and its treatment.

The two treatment options for pelvic AVM are surgical and endovascular. Controversy exists regarding the superior treatment for pelvic AVMs. AVM resection has a low success rate because of the high risk of severe intraoperative bleeding and damage to adjacent organs [2]. Furthermore, treatment by inflow artery ligation leads to recurrence owing to the development of collateral vessels and renders future catheterization difficult. Therefore, endovascular treatment has usually been performed for pelvic AVMs in recent years. In this case, the AVM was huge, and we considered that the AVM would not be successfully resected.

There has been a previous report of laparoscopic surgery for colorectal cancer with pelvic AVM; however, there are no reports of presurgical AVM embolization.

The surgical field may be restricted by dilated vessels, and AVMs can be damaged by accidental injury even if they do not originate from abdominal vessels, as seen in this case. Given the short operative time, minimal blood loss, and lack of postoperative complications in this case, preoperative embolization effectively reduces the risk of bleeding and enables a safe, minimally invasive procedure.

However, this technique requires advanced resources that may not be available at all facilities. Therefore, treatment plans should be tailored to the medical resources of the facility. In addition, there have been no reports of long‐term complications associated with IVR for pelvic AVM; however, long‐term follow‐up observation is required.

In conclusion, preoperative embolization of an asymptomatic pelvic AVM in a patient with sigmoid colon cancer may enable a safe, minimally invasive surgical procedure.

## Author Contributions

All authors contributed to data collection and the revision of this manuscript, and approved its submission.

## Ethics Statement

Patient anonymity was ensured. The principles of the Helsinki Declaration were respected in conducting the study.

## Consent

Written informed consent was obtained from this patient.

## Conflicts of Interest

Dr. Koichi Suda is an Editorial Board member of *ASES Journal* and a co‐author of this article. To minimize this conflicts of interest, he was excluded from all editorial decisions related to the acceptance of this article for publication. The other authors declare no conflicts of interest.

## Supporting information


**Figure S1.** Three‐dimensional computed tomography angiography of pelvic vessels (arteries). Inflow arteries branching off the left internal iliac artery (yellow arrowheads).

**Figure S2.** Three‐dimensional computed tomography angiography of pelvic vessels (nidus and vein). A dilated and tortuous nidus (red arrowheads) and outflow vein (yellow arrowheads) are seen on the right great saphenous vein.

**Video S3.** Digital subtraction angiography of the inflow artery before embolization. The nidus and outflow vein are contrasted from the inflow artery.

**Video S4.** Digital subtraction angiography of the inflow artery after embolization. The contrast agent remains in the nidus longer postembolization than before embolization. The inflow artery could not be completely embolized.

**Video S5.** Digital subtraction angiography of the outflow vein before embolization. The varicose vein of the outflow vein was punctured percutaneously. The nidus was contrasted retrogradely from the outflow vein.

**Video S6.** Angiography of the outflow vein after embolization. The outflow vein was embolized using penumbra and hydrogel coils. The coil was embolized until it could no longer be inserted. However, contrast agent leakage was observed. NBCA was used to perform additional embolization, and the vein was completely embolized.

**Figure S7.** Computed tomography image after arteriovenous malformation embolization. The dilated blood vessels have shrunk, and the contrast effect has disappeared (yellow arrowheads).

**Video S8.** Laparoscopic sigmoidectomy. During abdominal cavity observation, a T4a tumor was found in the sigmoid colon. No obvious dissemination was observed. The nidus was seen protruding into the abdominal cavity from the left retroperitoneum of the pelvis (3–12 s). Using an internal approach, the root of the inferior mesenteric artery, left colonic artery, and inferior mesenteric vein were clipped and dissected (13–26 s). The rectum was mobilized from the right side in the total mesorectal excision layer, and the autonomic nerves were preserved (27–32 s). Gentle mobilization was performed on the left side of the rectum while checking for the presence of embolized nidus (33–42 s). The assistant expanded the field of view so that the surgeon could identify the protruding nidus, and the mesentery was dissected to ensure that the energy device was not obscured (43–51 s). The rectum was transected to prevent the tip of the linear stapler from hitting the nidus. A linear stapler with absorbable suture reinforcement was used to enhance the hemostatic effect at the anastomosis (52 s–1 min 11 s). As the nidus on the dorsal side of the prostate gland was hard and elevated, a sizer was used before transanally inserting the circular stapler (1 min 12 s–1 min 18 s). A 25‐mm circular stapler was used to perform the anastomosis with the double‐stapling technique (1 min 19 s–1 min 28 s).

**Figure S9.** Surgical specimen. Cancer cell infiltration was absent (R0) at the resection margins.

## Data Availability

The data that support the findings of this study are available from the corresponding author upon reasonable request.
