# Supplementary figures and images for: Laparoscopic Sigmoidectomy in a Male Colon Cancer Patient With Pelvic Arteriovenous Malformation Using Preoperative Interventional Radiology: A Case Report
Source: Asian J Endosc Surg. 2025 Feb 20;18(1):e70037. doi: 10.1111/ases.70037 (PMC11842171; doi:10.1111/ases.70037)

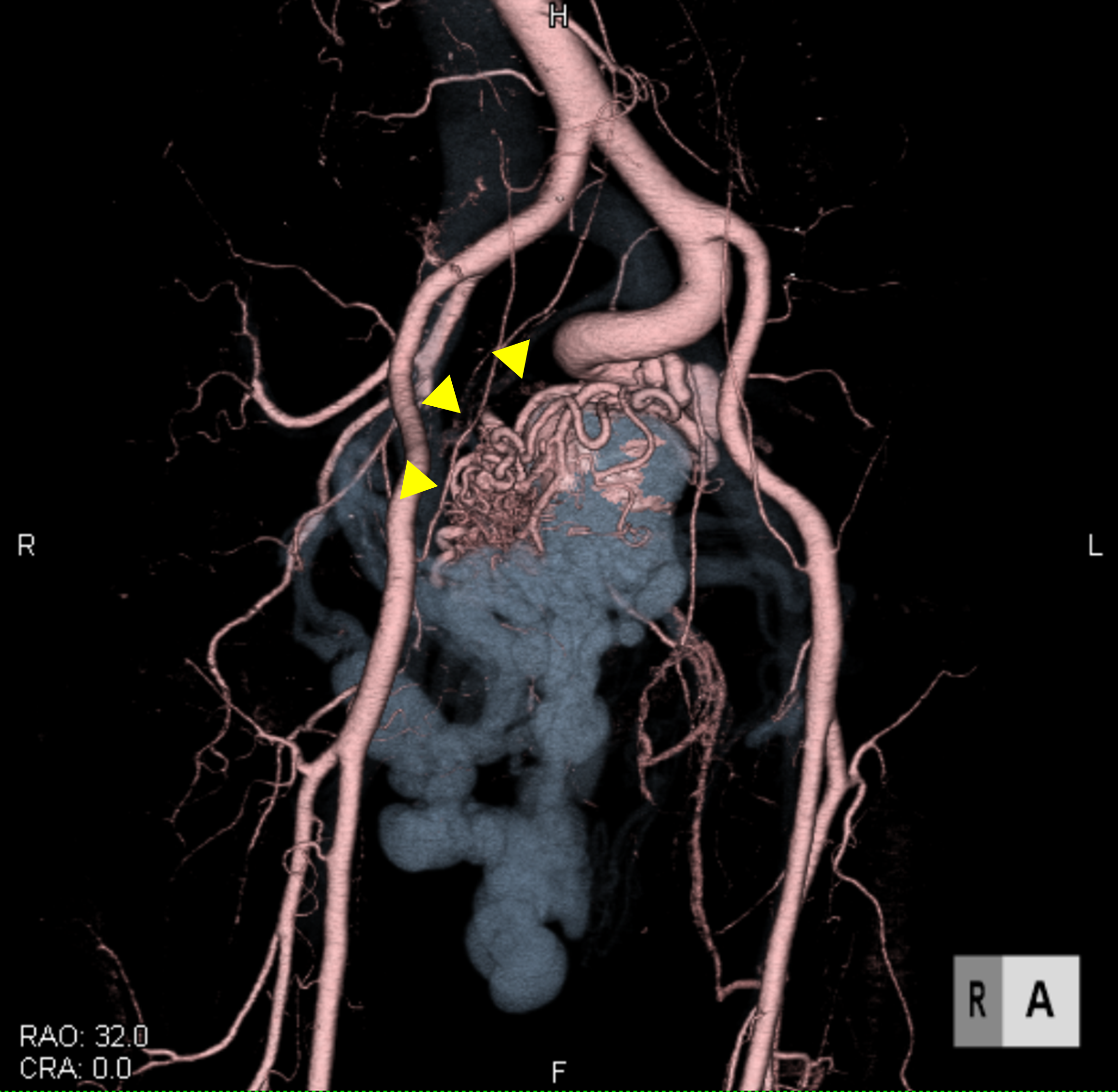

Supplement: Supplementary file 1 — Figure S1. Three‐dimensional computed tomography angiography of pelvic vessels (arteries). Inflow arteries branching off the left internal iliac artery (yellow arrowheads). [file ASES-18-e70037-s007.png]

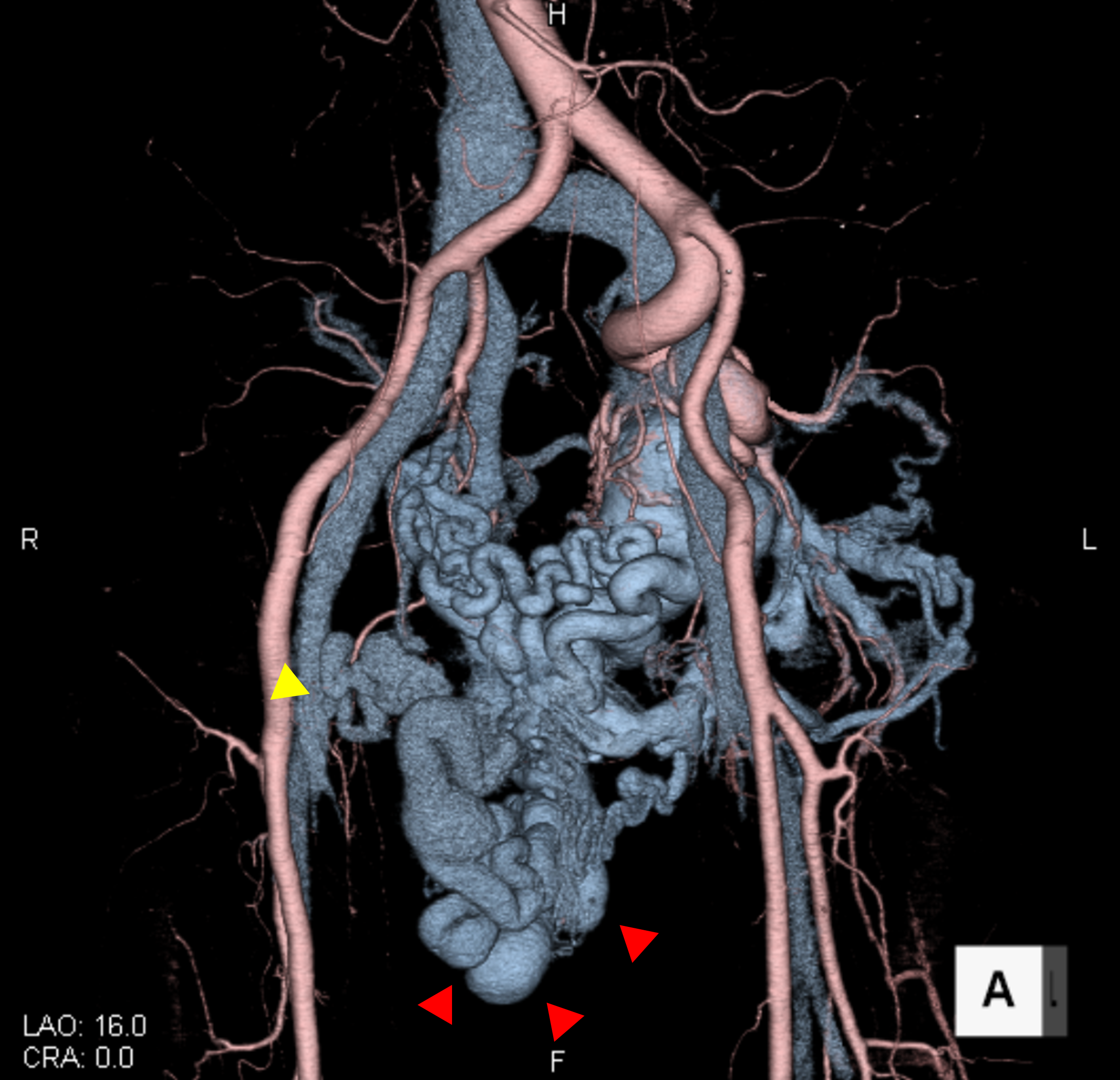

Supplement: Supplementary file 2 — Figure S2. Three‐dimensional computed tomography angiography of pelvic vessels (nidus and vein). A dilated and tortuous nidus (red arrowheads) and outflow vein (yellow arrowheads) are seen on the right great saphenous vein. [file ASES-18-e70037-s001.png]

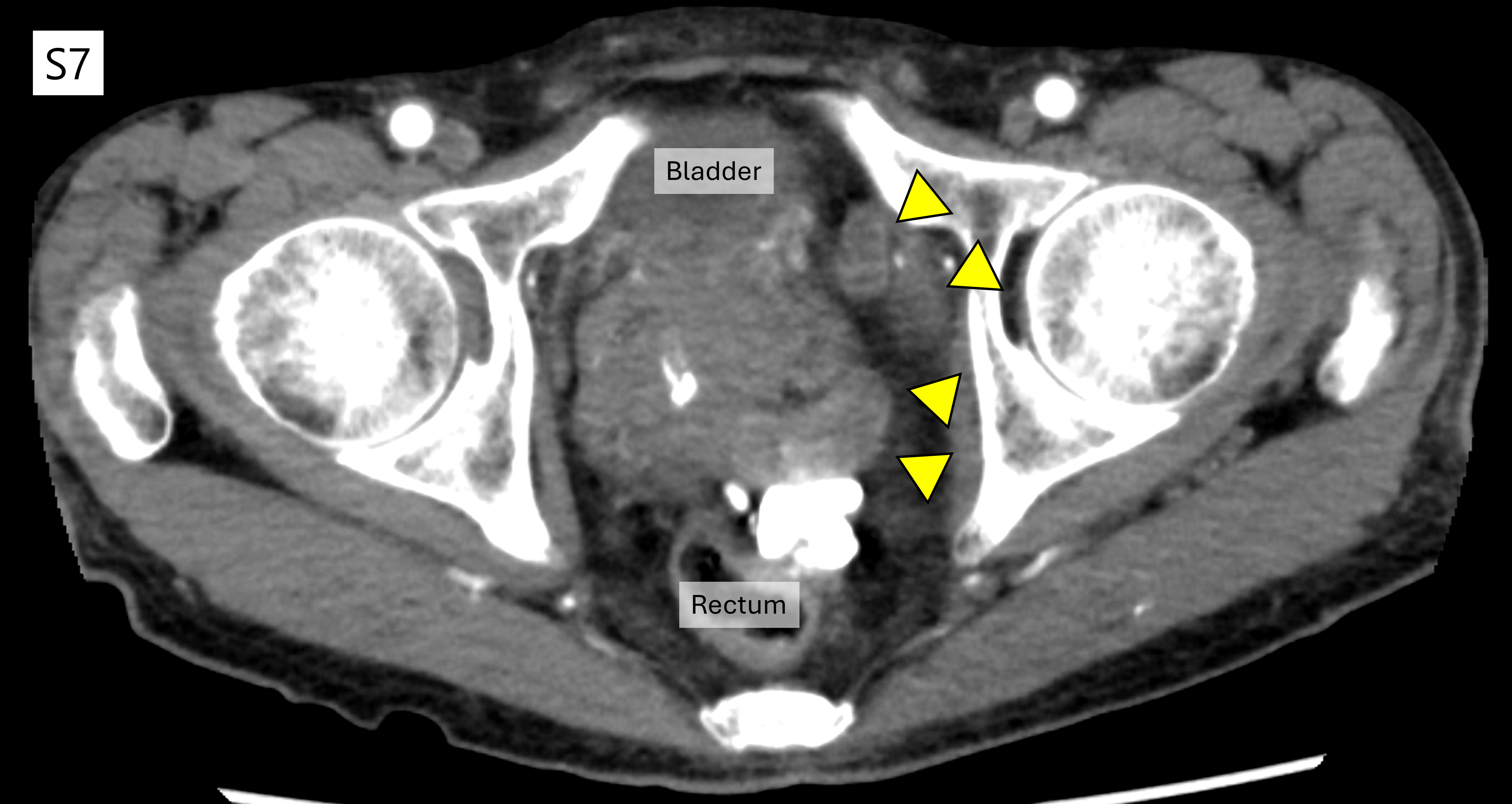

Supplement: Supplementary file 7 — Figure S7. Computed tomography image after arteriovenous malformation embolization. The dilated blood vessels have shrunk, and the contrast effect has disappeared (yellow arrowheads). [file ASES-18-e70037-s003.png]

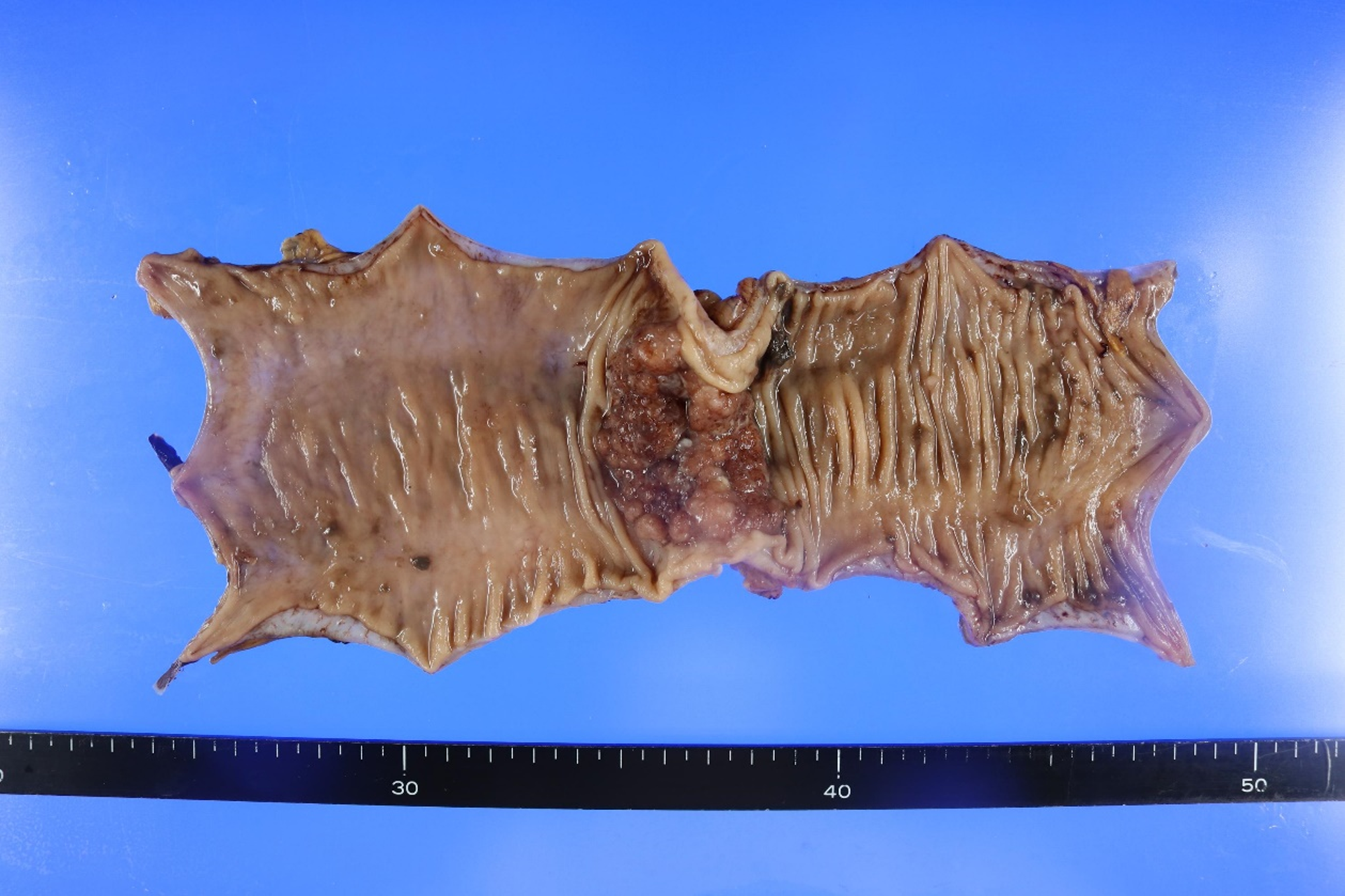

Supplement: Supplementary file 9 — Figure S9. Surgical specimen. Cancer cell infiltration was absent (R0) at the resection margins. [file ASES-18-e70037-s006.png]
